# Carie dentaire en milieu scolaire dans la ville de N’Djamena au Tchad : aspects épidémiologiques et habitudes bucco-dentaires chez des élèves âgés de 6 à 12 ans

**DOI:** 10.48327/mtsi.v4i2.2024.426

**Published:** 2024-05-02

**Authors:** Isidore MASSEDE, Stéphane MOUMBE TAMBA

**Affiliations:** 1 Centre de santé franco-anglais « La Lumière », section dentaire, N’Djamena, Tchad; 2 Université Lumière de Bujumbura, 643 Bujumbura, Burundi

**Keywords:** Dent, Carie dentaire, Prévalence, Facteurs associés, Pathologies bucco-dentaires, Enfant, École, Amtoukoui, Boutalbagara, École Prospérité mon Espoir, N’Djamena, Tchad, Afrique subsaharienne, Tooth, Dental caries, Prevalence, Associated factors, Oral pathologies, Children, School, Amtoukoui, Boutalbagara, École Prospérité mon Espoir, N’Djamena, Chad, Sub-Saharan Africa

## Abstract

**Introduction:**

La carie dentaire est une affection mondiale pouvant avoir des répercutions handicapantes. En Afrique, sa prévalence en milieu scolaire est très variable, due à la grande variabilité des habitudes alimentaires et d’hygiène bucco-dentaire. Cette étude vise à évaluer la prévalence de la carie dentaire, certains facteurs associés, et rechercher les pathologies bucco-dentaires associées à cette carie dans une circonscription de la ville de N’Djamena.

**Matériel et méthode:**

Il s’agit d’une étude transversale réalisée dans trois établissements du 7^e^ arrondissement de la ville de N’Djamena. Au total, 360 élèves âgés de 6 à 12 ans ont été recrutés entre octobre 2021 et septembre 2022. Les données personnelles des participants (âge, sexe, classe, professions des parents) ainsi que les résultats de l’examen bucco-dentaire auquel ces derniers avaient été soumis ont été collectés, puis analysés.

**Résultats:**

Au total, 185 élèves avaient au moins une dent cariée, soit une prévalence de 51,4 %. Parmi eux, 45 % possédaient au moins deux dents cariées. L’établissement scolaire fréquenté et le grignotage entre les repas étaient significativement associés à la présence de la carie (p<0,05). Les dents 36 (première molaire inférieure gauche) et 46 (première molaire inférieure droite) étaient majoritairement les plus attaquées par la carie (respectivement 21 % et 22 %). L’indice CAO mixte était de 0,6 et la fréquence globale des dents cariées (FGC) était égal à 51,9 %. Les moments de brossage, le matériel et les types de produits utilisés pour le brossage influençaient significativement l’apparition de la carie (p<0,05). Les participants présentant une dyschromie dentaire présentaient plus de caries dentaires.

**Conclusion:**

La maladie carieuse était bien présente dans les écoles ciblées et constituait un réel problème pour les élèves. La mise en place d’une politique de santé bucco-dentaire basée sur l’odontologie préventive par la sensibilisation des enfants et leurs parents contribuerait à la bonne formation des élèves.

## Abstract (en)

**Background:**

Dental caries is a global disease that can have disabling effects. In Africa, its prevalence in schools is very variable, due to the great variability of food habits and oral hygiene. This study aimed to assess the prevalence of dental caries, associated factors, and to research oral pathologies associated with that dental decay in one circumscription of the city of N’Djamena.

**Material and methods:**

This was a cross-sectional study conducted in 3 schools in the 7th borough of the city of N’Djamena. A total of 360 pupils aged between 6 and 12 years were recruited between October 2021 and September 2022. Each participant underwent to an oral examination which consisted in looking carefully at the pupil’s face (cheeks, lips) to note any deformities or possible facial asymmetry, and to check for any sensation of pain in any part of the face. In another hand, the oral examination aimed to look for any decay on all the teeth and determine the category and class of it. Finally, the personal data (age, sex, class, parents’ occupations) of each participant and the results of his oral examination were collected into a database and analyzed.

**Results:**

A total of 185 pupils had at least one decayed tooth, giving a prevalence rate of 51.4%. Among them, 45% had at least 2 decayed teeth. The school attended and snacking between meals were significantly associated with the presence of caries (p<0.05). Teeth 36 (lower left first molar) and 46 (lower right first molar) were the most often affected by caries (21% and 22% respectively). The mixt DMF index was 0.6 and the overall frequency of decayed teeth was 51.9%. According to Black’s classification, class II caries was the most prevalent (48%), while according to Baume’s classification, category II was the most prevalent (54%). The time of brushing, the material and the type of product used significantly influenced the appearance of caries (p<0.05). Participants with dental dyschromia had more tooth decay.

**Conclusion:**

Caries was prevalent in the targeted schools and represented a real problem for pupils. Implementing an oral health policy based on preventive dentistry by raising awareness among children and their parents would contribute to the proper education of pupils.

## Introduction

La carie dentaire est une pathologie infectieuse des dents caractérisée par un processus chimico-parasitaire provoquant la déminéralisation de l’émail, ensuite sa destruction. Il s’agit d’une maladie buccale d’origine bactérienne, les streptocoques mutants étant considérés comme les principales bactéries à l’origine de son apparition [15]. Elle est l’une des pathologies les plus fréquentes au niveau de la sphère orale et on estime que deux milliards de personnes souffrent de caries des dents définitives et 520 millions d’enfants, de caries des dents de lait [16]. Après l’éruption dentaire chez les enfants, si l’hygiène bucco-dentaire n’est pas bien observée, et par l’influence des facteurs notamment sociaux, environnementaux, et ceux liés au mode de vie, la dent se déminéralise et il se forme une cavité appelée la carie dentaire [4].

En Afrique, les prévalences des affections bucco-dentaires, dont la carie chez les enfants en milieu scolaire, sont disparates et élevées selon les pays et les auteurs (58,2 % en Afrique du Sud, 33,7 % au Swaziland, 75 % au Gabon, 31 % à Zanzibar, 87,2 % en Côte d’Ivoire) [5,9,10,17,21]. L’absence d’hygiène buccale, combinée aux changements d’habitudes alimentaires, surtout en zone urbaine, chez un nombre considérable d’enfants, semble être la cause de l’ampleur de cette pathologie asymptomatique au niveau de l’émail, mais qui, atteignant la pulpe, devient plus douloureuse. La douleur causée par cette pathologie handicape souvent le bon apprentissage des élèves en les empêchant de bien suivre les cours, voire en s’absentant de l’école.

Les facteurs tels que l’hérédité et l’hygiène jouent un rôle capital dans la formation de la carie, mais l’alimentation demeure le facteur favorisant le plus important.

La carie dentaire constitue donc un véritable problème de santé publique et communautaire, réduisant la capacité de bien étudier, travailler, socialiser, parler, et manger, entraînant aussi une diminution de la qualité de vie des élèves.

Dans le contexte tchadien, la carie dentaire est une affection bien connue et présente dans la population. La création de l’Association stop carie dentaire (ASCAD) en 2016 est un pas considérable dans la lutte communautaire contre cette affection. Cependant, aucune étude n’a été réellement menée pour faire un état des lieux spécialement dans la ville de N’Djamena à notre connaissance. C’est ainsi dans le but d’apporter une contribution scientifique sur la question que nous avons décidé d’entreprendre ce travail de recherche qui avait pour principal objectif de déterminer la prévalence de la carie, ses facteurs favorisants et les pathologies bucco-dentaires associées à cette carie chez les élèves de 6 à 12 ans en milieu scolaire.

## Matériel et méthodes

### Cadre et période d’étude

Il s’agit d’une étude transversale réalisée sur une année, d’octobre 2021 à septembre 2022, dans la ville de N’Djamena, capitale du Tchad. La ville de N’Djamena est subdivisée en 10 arrondissements comprenant chacun plusieurs écoles primaires, soit publiques, soit privées. Pour des exigences logistiques, notre étude s’est déroulée essentiellement dans le 7^e^ arrondissement de la ville. Le choix du 7^e^ arrondissement de la ville de N’Djamena comme lieu d’étude a été motivé par sa multiculturalité. En effet, les habitants de cet arrondissement appartiennent aux différentes couches sociales et on peut également y rencontrer une majorité des différents groupes ethniques du pays. Trois établissements primaires de cet arrondissement, choisis selon leur composition sociale et le style de vie général des élèves qui les fréquentent, ont été retenus pour cette étude. Il s’agissait de deux écoles publiques dont l’école officielle d’Amtoukoui comprenant 4 101 élèves à majorité musulmane et de niveau de vie aisé et l’école officielle de Boutalbagara avec 2 264 élèves à majorité chrétienne avec un niveau de vie bas. Le troisième établissement était l’école Prospérité mon Espoir, une école privée dont les élèves qui la fréquentent sont assez variés en termes de religion, avec un niveau de vie moyen et un effectif total de 319 élèves.

### Échantillonnage

Cette étude a concerné des élèves âgés de 6 à 12 ans, tout sexe confondu, appartenant aux établissements scolaires sélectionnés. Afin d’obtenir un échantillon représentatif, nous avons utilisé la formule de Lorentz pour calculer la taille minimale de l’échantillon. Cette formule tient compte du niveau de confiance admis, de la proportion estimée de la population qui présente la caractéristique (carie dentaire) ainsi que de la marge d’erreur tolérée. Ainsi, nous avons utilisé la prévalence de 75 % de la carie rapportée dans une étude similaire réalisée dans la sous-région, au Gabon [10]. Après calcul, nous avons trouvé une taille minimale d’échantillon représentatif de 288 participants (avec un niveau de confiance à 95 % et une marge d’erreur de 5 %). En définitive, nous avons décidé d’enrôler pour notre étude, un total de 360 participants, au vu du matériel à notre disposition. Tenant en considération le nombre de salles de classe dans les écoles, nous avons décidé d’enrôler 150 élèves dans chacune des écoles publiques et 60 dans l’école privée.

### Sélection des participants et collecte des données

Des contacts avaient été préalablement établis avec les directeurs d’écoles afin de leur expliquer les fins de l’étude, d’obtenir leur autorisation et de convenir de la faisabilité, du déroulement de l’enquête et de la collecte des données. Plusieurs visites ont été effectuées dans les écoles au cours de l’année. Ce sont les responsables des établissements qui, après avis favorable des parents, se sont occupés de rassembler les enfants durant certains jours de classe afin qu’ils suivent avant tout un exposé sur l’hygiène bucco-dentaire. À la suite de cet exposé, une invitation à réaliser un examen bucco-dentaire et à répondre à certaines questions a été adressée aux enfants. Les élèves portés volontaires (sans distinction d’avoir préalablement un éventuel problème bucco-dentaire ou pas) devaient juste lever la main puis, accompagnés des responsables de l’établissement, se rendre dans une des salles de l’école préalablement arrangée pour servir de salle d’attente. Les données récoltées au cours de cette étude ont premièrement été obtenues après une anamnèse de chaque participant dans la salle d’attente. Grâce à un questionnaire expressément conçu pour l’étude, les questions posées à chacun des élèves ont permis de relever son âge, sa classe, la profession de ses parents, ses habitudes alimentaires (nombre de repas, grignotage entre les repas ou non) et ses habitudes en matière d’hygiène bucco-dentaire (fréquence de brossage, matériels utilisés au cours des brossages). Au cours de ce questionnement, les élèves dont l’âge ne correspondait pas à la tranche définie ou ceux avec des réponses très incertaines ou encore avec des difficultés à s’exprimer ont été exclus de l’étude.

Après le remplissage du questionnaire, chaque enfant devait se rendre dans la deuxième salle aménagée avec des fauteuils dentaires portables et des plateaux techniques pour l’examen bucco-dentaire. Cet examen s’est déroulé en deux étapes :

- étape exobuccale, qui a consisté à regarder le visage de l’élève (les joues, les lèvres) pour noter toutes les déformations ou une probable asymétrie faciale, suivie d’une palpation permettant d’apprécier la surface cutanée, de noter une sensation de douleur dans une partie du son visage;
- étape endobuccale, durant laquelle l’élève a ouvert grandement la bouche, et où toutes les dents ont été soigneusement observées, à l’aide d’une sonde dentaire et du miroir buccal, pour rechercher d’éventuelles caries sur toutes les dents, tant sur les dents définitives que sur les dents de lait. En présence de carie, la détermination de sa catégorie et de sa classe a tout de suite été effectuée selon la classification de Black et de Baume, puis mentionnée dans la fiche individuelle de chaque participant. À la fin de cet examen, des conseils en matière d’hygiène bucco-dentaire ont à nouveau été prodigués aux élèves en fonction du résultat observé.

Après l’examen de chaque élève, le matériel utilisé a été décontaminé avec de l’eau chlorée, ensuite lavé à l’eau, puis séché et acheminé dans une clinique dentaire pour la stérilisation dans un stérilisateur Poupinel en vue d’une utilisation ultérieure.

Les visites dans les écoles pour collecter les données se sont poursuivies pendant plusieurs jours en fonction de la disponibilité des établissements, jusqu’à ce que le nombre d’enfants reçus dans chaque école atteigne le quota défini pour chacune des écoles sélectionnées.

### Classification des dents cariées

Les dents cariées identifiées dans notre étude ont été réparties selon les classifications de Black et de Baume. La classification de Black [11] concerne le siège de la lésion carieuse et comprend cinq classes :

- Classe I : carie au niveau des défauts de structure dans les puits et sillons;
- Classe II : carie proximale des prémolaires et des molaires;
- Classe III : carie proximale des incisives et canines sans atteinte des bords incisifs;
- Classe IV : carie proximale des incisives et canines avec atteinte des bords incisifs;
- Classe V : carie des collets dentaires.

La classification de Baume [3] en revanche, est une classification clinique décrivant la symptomatologie pulpaire et les douleurs ressenties par les patients. Elle permet de fournir des indications précises sur le traitement à mettre en place. Cette classification comprend quatre catégories :

- Catégorie I : pulpe vivante, asymptomatique, pouvant être protégée par coiffage pulpaire;
- Catégorie II : pulpe vivante symptomatique, la vitalité pulpaire peut être conservée à l’aide d’un coiffage ou d’une pulpotomie.;
- Catégorie III : pulpe vivante dont l’indication est la bio-pulpectomie, pour causes esthétiques, iatrogènes, prothétiques ou pronostiques;
- Catégorie IV : pulpe nécrosée associée à une atteinte infectieuse de la dentine radiculaire, complications péri-apicales souvent associées.

### Saisie et analyse des données

Les données obtenues à la suite des questions posées et de l’examen bucco-dentaire de chaque participant ont été compilées sur une base sur Microsoft Excel 2016. Ces données ont ensuite été analysées grâce au Logiciel SPSS 26. La statistique descriptive avec la détermination des pourcentages et la réalisation des graphiques a été effectuée avec Microsoft Excel 2016. L’analyse statistique a comporté le test de khi-deux pour déterminer les différentes associations entre variables qualitatives et le test-t de Student avait été utilisé pour comparer les moyennes. Les résultats étaient considérés comme statistiquement significatifs pour une valeur de p < 0,05.

### Considérations éthiques

La réalisation de l’étude et la collecte des données ont été faites après obtention de l’autorisation des responsables de chacun des établissements ciblés ainsi que des parents des élèves. L’anonymat des participants ainsi que la confidentialité des données collectées et traitées ont été strictement respectées.

## Résultats

### Caractéristiques générales de l’échantillon (Tableau I)

**Tableau I:** Caractéristiques générales de l’échantillon General characteristics of the sample

	École officielle d’Amtoukoui		École officielle de Boutalbagara		École Prospérité mon Espoir		Total	
	Effectif	%	Effectif	%	Effectif	%	Effectif	%
**Sexe**								
féminin	67	37	81	45	33	18	181	50,3
masculin	83	46	69	39	27	15	179	49,7
**Âge (ans)**								
6	19	45	16	38	7	17	42	11
7	10	39	11	42	5	19	26	7
8	17	50	9	27	8	24	34	9
9	15	46	12	36	6	18	33	9
10	34	55	17	27	11	18	62	17
11	22	49	14	31	9	20	45	12
12	33	28	71	60	14	12	118	33
**Niveau scolaire**								
CPa I	28	42	28	42	10	15	66	18
CPa II	28	58	11	23	9	19	48	13
CEb I	14	24	36	61	9	15	59	16
CEb II	37	64	10	17	11	19	58	16
CMc I	23	44	21	40	8	15	52	14
CMc II	20	26	44	57	13	17	77	21
**Profession du parent ou tuteur**								
commerçant	37	40	39	43	16	17	92	26
libre emploi	42	39	58	53	9	8	109	30
entrepreneur	9	69	1	8	3	23	13	4
militaire	12	52	5	22	6	26	23	6
travailleur dans le secteur public ou privé	50	40	47	38	26	21	123	34
**Grignotage entre les repas**								
oui	134	44,2	124	40,9	45	14,9	303	84,2
non	16	28,1	26	45,6	15	26,3	57	15,8

a : cours préparatoire; b : cours élémentaire; c : cours moyen

De façon globale, le sex-ratio de notre échantillon était de 0,9 et la moyenne d’âge des participants était de 9,8±2,1 ans. Il n’y avait pas de différence significative d’âge entre les filles et les garçons de notre échantillon (9,9±2,2 ans contre 9,8±2 ans, p = 0,719). Les enfants âgés de 12 ans et ceux au CMII étaient les plus nombreux (respectivement 33 % et 21 %).

### Prévalence de la carie dentaire et facteurs associés (Tableau II)

**Tableau II:** Répartition des facteurs sociodémographiques associés à la présence de la carie dentaire Distribution of socio-demographic factors associated with the presence of dental caries

	Oui		Non		p
	Effectif	%	Effectif	%	
**Sexe**					
féminin	91	50	90	50	0,671
masculin	94	53	85	47	
**Âge (ans)**					
6	23	55	19	45	0,822
7	13	50	13	50	
8	17	50	17	50	
9	18	55	15	45	
10	34	55	28	45	
11	26	58	19	42	
12	54	46	64	54	
**Établissements scolaires**					
école Prospérité mon Espoir	18	30	42	70	<0,0001*
école Officielle d’Amtoukoui	94	63	56	37	
école Officielle de Boutalbagara	73	49	77	51	
**Profession du parent ou tuteur**					
commerçant	48	52	44	48	0,713
libre emploi	55	50	54	50	
entrepreneur	6	46	7	54	
militaire	15	65	8	35	
travailleur dans le secteur public ou privé	61	50	62	50	
**Grignotage entre les repas**					
oui	183	60	120	40	<0,0001*
non	2	4	55	96
**Moyenne d’âge des élèves (ans)**	9,7±2,1	9,9±2,1	0,449

* Significatif

La présence de la carie dentaire a été évaluée chez les participants à la suite d’un minutieux examen bucco-dentaire. Au total, parmi les 360 élèves retenus dans notre étude, 185 d’entre eux avaient au moins une dent cariée, soit une prévalence de 51,4 %.

**Figure 1:**
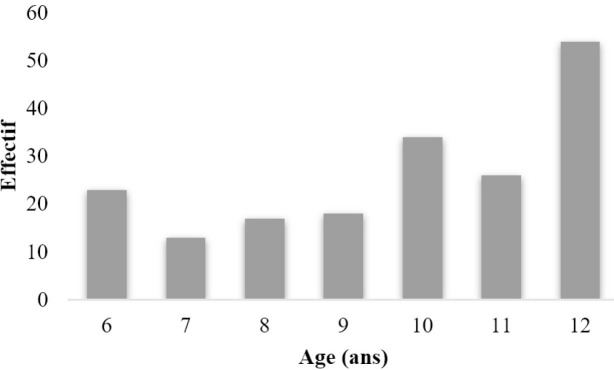
Évolution des élèves avec caries dentaires en fonction de l’âge Evolution of pupils with dental caries according to age

Les élèves âgés de 12 ans, 10 ans et 6 ans avaient respectivement les taux de caries dentaires les plus élevés dans notre série (Fig. 1). Parmi les facteurs recherchés, l’établissement scolaire fréquenté par les élèves et le grignotage entre les repas étaient significativement associés à la présence de la carie dentaire chez les participants.

### Nombre de dents cariées (Fig. 2)

**Figure 2:**
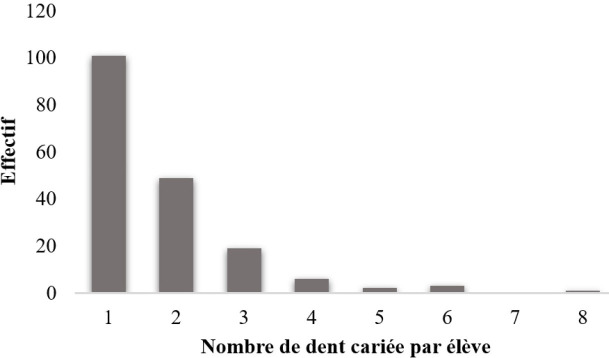
Distribution du nombre de dent cariée par élève Distribution of the number of decayed teeth per pupil

Au total, nous avons observé 316 dents cariées chez les 185 élèves présentant au moins une carie dentaire et le nombre de dents cariées a été déterminé chez chacun d’entre eux.

Parmi les élèves, 101 (54,6 %) d’entre eux ne possédaient qu’une seule dent cariée et les 84 autres (45 %) en possédaient au moins deux. Cependant, un participant en possédait jusqu’à huit.

L’examen bucco-dentaire a révélé que les dents 36 (première molaire inférieure gauche) et 46 (première molaire inférieure droite) étaient majoritairement les plus attaquées par la carie dentaire, avec respectivement 21 % et 22 % des cas.

### Indice CAO mixte et fréquence globale des dents cariées (FGC)

Les données recueillies dans notre travail nous ont également permis de calculer l’indice CAO mixte (pour les deux types de dents : permanentes et lactéales) et la FGC selon les paramètres suivants :

- nombre de dents cariées (C) = 206;
- nombre de dents absentes (A) = 2;
- nombre de dents obturées (O) = 0;
- nombre de dents CAO mixte = 208;
- nombre de sujets avec les dents cariées = 185;
- nombre des sujets avec des dents CAO mixte =187;
- nombre de sujets examinés = 360.

Ainsi, l’indice CAO mixte était égal à 0,6 et la FGC était égal 51,9 %.

### Classification des dents cariées (Tableau III)

**Tableau III:** Classification des dents cariées rencontrées Classification of decayed teeth

Classification	Effectif	%
**Classification de Black**		
Classe I	84	27
Classe II	151	48
Classe III	41	13
Classe IV	21	7
Classe V	19	6
total	316	100
**Classification de Baume**		
Catégorie I	122	39
Catégorie II	171	54
Catégorie III	18	6
Catégorie IV	5	1
total	316	100,0

Selon la classification de Black, la carie de classe II était la plus présente (48 %) tandis que selon la classification de Baume, la catégorie II était la plus présente (54 %).

### Hygiène bucco-dentaire et pathologies associées (Tableau IV)

**Tableau IV:** Hygiène bucco-dentaire et pathologies associées Oral hygiene and associated pathologies

	Enfants avec caries		Enfants sans caries		p
	Oui		Non		
	Effectif	%	Effectif	%	
**Succion digitale**	**17**	**9**	**4**	**2**	0,005*
**Fréquence de brossage quotidien**					
matin	97	52	117	67	0,005*
midi	5	3	3	2	0,524
soir	0	0	1	1	0,303
matin-midi	0	0	2	1	0,303
matin-soir	18	10	20	11	0,600
matin-midi-soir	7	4	3	2	0,232
plus de trois fois	14	8	11	6	0,632
pas de brossage	44	24	18	10	0,0007*
**Matériel de brossage utilisé**					
brosse à dent	111	60,0	141	80,6	< 0,0001*
siwak**	21	11	16	9	0,490
brindille de bois	5	3	2	1	0,284
doigts	3	2	2	1	0,698
aucun	45	24	14	8	< 0,0001*
**Produits utilisés pour l’hygiène bucco-dentaire**					
pâte dentifrice	109	58,9	144	82,3	< 0,0001*
charbon	5	3	4	2	0,800
savon	3	2	3	2	0,945
cendres	5	3	1	1	0,114
bicarbonate de sodium	2	1	0	0	0,168
aucun	61	33	23	13	< 0,0001*
**Pathologies liées à l’hygiène bucco-dentaire**					
biocom (biofilm ou plaque dentaire)	20	11	5	3	0,003*
dyschromie	2	1	0	0	0,168
fluorose	1	1	2	1	0,530
halitose	0	0	1	1	0,303
malocclusion	1	1	1	1	0,966
tartre	5	3	15	9	0,015*
aucune	156	84,3	151	86,3	0,600

* Significatif

** Le siwak, également appelé miswak, est une racine provenant de l’arbuste *Salvadora persica,* dit aussi « arbre brosse-dents ». Il est utilisé par certaines personnes pour se brosser les dents.

L’hygiène bucco-dentaire a été évaluée ainsi que les pathologies liées à cette hygiène.

Les élèves avec succion digitale présentaient significativement plus de caries dentaires. Concernant la fréquence de brossage quotidien, les élèves se brossant seulement le matin présentaient significativement moins de caries, tandis que ceux qui ne se brossaient pas présentaient significativement plus de caries. S’agissant du matériel et produits de brossage, les élèves utilisant la brosse à dent et ceux utilisant la pâte dentifrice présentaient moins de carie tandis que ceux n’utilisant aucun matériel ni produit pour le brossage présentaient plus de caries. Dans la distribution des pathologies liées à cette hygiène, les élèves présentant des plaques dentaires avaient significativement plus de caries dentaires.

## Discussion

Le problème de santé publique que représente la carie dentaire est assez connu de façon globale. Malgré cela, l’absence des données référencées sur ce sujet dans notre contexte a constitué le principal motif pour la réalisation de cette étude. Il s’agit d’une étude pionnière qui visait avant tout à faire un état des lieux en ce qui concerne l’affection carieuse chez les élèves d’une circonscription bien précise de la ville de N’Djamena au Tchad. Nous avons pu faire ressortir au cours de notre travail une prévalence de la carie dentaire de 51,4 % et avons pu retrouver que l’établissement scolaire fréquenté et le grignotage entre les repas étaient significativement associés à la présence de cette carie. L’examen bucco-dentaire minutieux réalisé a également permis de relever que les dents 36 et 46 étaient majoritairement les plus attaquées par la carie et que les élèves présentant des plaques dentaires avaient significativement plus de caries dentaires. Dans la même lancée que d’autres études africaines, les résultats retrouvés dans notre travail permettent d’apprécier l’ampleur du problème de la carie dentaire dans notre contexte. Dans cette étude, nous nous sommes seulement concentrés sur un arrondissement parmi les 10 qui composent la ville de N’Djamena : nul doute que la prévalence de la carie dentaire retrouvée ici pourrait varier d’un arrondissement à un autre et la prévalence générale dans la ville en serait affectée. En outre, les élèves ayant accepté de participer à l’étude étaient tous volontaires. Il y a matière à penser que ce sont ceux qui avaient déjà un soupçon de problème dans la bouche qui se sont plus portés volontaires (bien que l’invitation eût été lancée à tous, avec ou sans problème bucco-dentaire préalable) d’où une telle prévalence élevée. D’autres facteurs, tels que la peur ou la timidité des élèves, ne sont pas à exclure dans le conditionnement du choix de participer à l’étude ou pas. Ces biais et limites ont certainement eu une influence sur l’échantillon de notre travail et, par conséquent, sur l’ensemble des résultats trouvées dans notre étude. Cependant, il n’en demeure pas moins que cette étude pionnière a permis d’avoir une idée générale du problème en fournissant des données préliminaires qui pourraient orienter une politique publique de lutte contre ce fléau, spécialement chez les enfants.

La tranche d’âge de 6 à 12 ans ciblée dans ce travail représente un groupe où les enfants apprennent à devenir indépendants et commencent à prendre des habitudes qui sont appelées à perdurer. Au cours de notre enquête, le sexe féminin était, de peu, plus présent. D’autres auteurs tels que Ayebameru *et al.* au Nigeria en 2021 [2] ont également rapporté des prédominances féminines au cours de leurs enquêtes chez des enfants âgés de 9 à 12 ans (53 %).

Plus de la moitié de notre échantillon avait au moins une dent cariée (51,4 %). Cette proportion assez élevée était toutefois inférieure à celle de Koko *et al.* au Gabon en 2009 qui avaient trouvé une prévalence de 75 % [10].

Cependant, la prévalence retrouvée dans notre étude reste supérieure à celles rapportées par Petersen *et al.* à Zanzibar en 1998 [17], avec une prévalence de 31 %, et par Dieng *et al.* en 2022, avec une prévalence de 47,4 % [6]. Ces différences de prévalences pourraient s’expliquer par la différence de styles de vie et d’habitudes en termes d’hygiène bucco-dentaire dans les différents groupes étudiés. Ceci concorde avec la littérature qui fait part d’une grande variation en termes de distribution de la carie dentaire. L’analyse des résultats n’a révélé aucune association significative entre la survenue de la carie dentaire et le sexe des élèves. Ce résultat contraste avec celui de Diombana *et al.* au Mali en 1990 [8] qui ont trouvé que la carie était liée au sexe et que l’activité carieuse des filles était légèrement plus élevée que celle des garçons. Par ailleurs, d’autres auteurs [20], n’ont pas retrouvé d’influence significative de l’âge dans l’apparition de la carie dentaire, tout comme dans ce travail. Cependant, Maiga, dans ses travaux menés au Mali en 2021 [12], a trouvé un résultat discordant. En effet, dans sa série, l’âge influençait la survenue de la carie dentaire et les élèves de six ans étaient les plus atteints. Dans un autre sens, Dieng *et al.* [6] montraient plutôt une tendance à la diminution de la proportion de dents cariées chez les enfants de 12 ans comparés à ceux de 7 ans. Cette discordance sur l’influence de l’âge dans la survenue de l’affection carieuse soulève la problématique de l’âge à laquelle une bonne hygiène bucco-dentaire est mise en place. En effet, dans ce travail, nous avons montré l’association significative entre la fréquence de brossage, le matériel ainsi que le produit de brossage et la survenue de la carie dentaire.

Concernant la distribution de la carie selon les écoles, la plus grande proportion de carie a été retrouvée dans l’école officielle d’Amtoukoui, majoritairement fréquentée par des élèves de niveau de vie plus aisé que les élèves des autres écoles. Ce constat est à mettre en parallèle avec le fait que cette école avait aussi un taux de grignotage plus élevé que les autres. En effet, le grignotage ainsi que le type d’aliments grignotés par les enfants variaient selon les écoles, les quartiers et les ressources des parents. En outre, le grignotage était significativement associé à la survenue de la carie dentaire chez les participants. Les élèves atteints de carie avaient plus l’habitude de grignoter entre les repas. Ces résultats montrent un susceptible lien entre niveau de vie aisé, grignotage et apparition de la carie dentaire. Ceci pourrait s’expliquer par la présence de sucres fermentescibles dans les aliments fréquemment consommés par les enfants (sucettes, biscuits, chocolats…) qui sont des facteurs essentiels dans la survenu de la carie.

Cependant, l’absence de différence significative entre la profession des parents ou tuteurs et la présence de carie chez les élèves semble indiquer que le niveau de vie des familles n’est pas directement lié à la profession déclarée des parents. Toutefois, certaines études ont parfois rapporté une prévalence de la carie significativement plus élevée, soit dans les familles où les parents étaient ouvriers ou chômeurs [7], soit selon le degré de précarité matérielle de la famille ou la classe sociale du chef de famille [14, 18].

L’examen bucco-dentaire réalisé chez les participants a permis de relever que les premières molaires permanentes mandibulaires (36 et 46) étaient les plus cariées. Tamba-Fall *et al.* au Sénégal en 2011 [19], dans une étude sur quatre cas, ont fait état d’une prédominance des molaires parmi les dents cariées, tandis que Aidara et Bourgeois, au Sénégal en 2014 [1], ont rapporté dans leur travail que les premières molaires mandibulaires étaient plus sensibles à la carie dentaire chez les écoliers sénégalais de 12 ans et de 15 ans. Bien que ces études ne soient pas directement comparables à la nôtre, elles vont néanmoins dans le même sens dans le fait que les dents 36 et 46 sont les plus atteintes.

L’évaluation de l’hygiène bucco-dentaire des élèves représente un point fondamental dans cette étude. En effet, les élèves présentant des plaques dentaires avaient significativement plus de caries dentaires. Il est connu que si l’hygiène bucco-dentaire n’est pas efficace, les débris alimentaires qui se trouvent dans les puits, les sillons et les fossettes dentaires peuvent créer une cavité sur les dents au cours du temps sous l’influence des bactéries cariogènes. Ce constat dans notre travail est proche de celui de Majoli *et al.* au Cameroun en 2006 [13] qui rapportent que la fréquence du brossage des élèves était insuffisante pour assurer une bonne hygiène bucco-dentaire. L’insuffisance de cette hygiène est reflétée par la grande proportion des élèves de notre série ayant des pathologies liées à l’hygiène buccodentaire et à l’association significative entre la présence de ces pathologies et la survenue de la carie dentaire.

Malgré les limites qu’elle comporte, cette étude donne une estimation de la situation de l’hygiène bucco-dentaire dans notre contexte. D’autres projets de plus grande envergure pourraient être envisagés avec un échantillonnage aléatoire dans toute la ville en vue d’avoir des données plus fiables et plus représentatives afin de pouvoir établir des campagnes de sensibilisation et de lutte contre la carie dentaire et les pathologies buccodentaires à N’Djamena en particulier et au Tchad en général.

## Conclusion

La maladie carieuse est un véritable problème de santé publique en général et des milieux scolaires, en particulier dans les écoles tchadiennes qui ont pris part à cette étude. Ce travail réalisé dans le 7^e^ arrondissement de la ville de N’Djamena visait à évaluer la prévalence de la carie dentaire dans des milieux scolaires, les facteurs et pathologies associés, ainsi que le comportement des participants vis-à-vis de l’hygiène buccodentaire. La carie dentaire était bien présente avec une forte prévalence parmi les participants, les dents 36 et 46 étant les plus affectées. Notre étude a aussi révélé une précarité de l’hygiène bucco-dentaire chez les élèves avec un non-respect des fréquences de brossage approprié et l’utilisation de matériels et de produits non adaptés. Tous ces éléments se sont avérés significativement associés à l’apparition de la carie dentaire. Les dents cariées étaient majoritairement de catégorie II, avec une atteinte de la dentine rendant ainsi les dents plus sensibles et douloureuses. Cette douleur et sensibilité éprouvées pourraient constituer autant d’éléments susceptibles d’handicaper les élèves et influencer leurs rendements scolaires. Devant cette situation, une nouvelle orientation de la politique de santé bucco-dentaire basée sur l’odontologie préventive s’impose pour la bonne formation des élèves dans les écoles primaires de N’Djamena et du Tchad.

## Contribution des auteurs

Massede I. a dirigé l’étude depuis sa conception, le protocole de recherche, la collecte des données, la rédaction et la révision du manuscrit. Moumbe Tamba S. a révisé le protocole, effectué l’analyse statistique des données, la rédaction et révision du manuscrit. Tous les auteurs ont lu et approuvé le manuscrit final.

## Remerciements

Les auteurs tiennent à présenter leur gratitude à tous les responsables des groupes scolaires ciblés, aux parents et aux enfants qui ont accepté de collaborer et de participer à l’étude. Nous remercions également le personnel de santé qui a aidé dans l’évaluation buccodentaire.

## Liens d’intérêts

Les auteurs déclarent ne pas avoir de liens d’intérêts.
